# Identification of differentially methylated regions associated with both liver fibrosis and hepatocellular carcinoma

**DOI:** 10.1186/s12876-024-03149-3

**Published:** 2024-02-01

**Authors:** Suguru Kurokawa, Takuro Kobori, Masato Yoneda, Yuji Ogawa, Yasushi Honda, Takaomi Kessoku, Kento Imajo, Satoru Saito, Atsushi Nakajima, Kikuko Hotta

**Affiliations:** 1 Laboratoy of Pathophysiology and Pharmacotherapeutics, Faculty of Pharmacy, Osaka Ohtani University, 3-11-1 Nishikiori-kita, Tondabayashi, Osaka, 584-8540 Japan; 2 Department of Gastroenterology and Hepatology, Yokohama City University Graduate School of Medicine, 3-9 Fukuura, Kanazawa-ku, Yokohama, Kanagawa 236-0004 Japan; 3 Department of Gastroenterology, National Hospital Organization Yokohama Medical Center, 3-60-2 Harajyuku, Totsuka, Yokohama, 245-8675 Japan; 4 Department of Palliative Medicine, International University of Health and Welfare Narita Hospital, 852, Hatakeda, Narita, 286-8520 Japan; 5 Department of Gastroenterology, Shin-yurigaoka General Hospital, 255 Furusawatsuko, Asao, Kawasaki, 2150-0026 Japan

**Keywords:** Epigenetics, Cirrhosis, Hepatocellular carcinoma, *ZBTB38*, *ZC3H3*, *FOXK1*, *KAZN*

## Abstract

**Background:**

Liver fibrosis is a major risk factor for hepatocellular carcinoma (HCC). We have previously reported that differentially methylated regions (DMRs) are correlated with the fibrosis stages of metabolic dysfunction-associated steatotic liver disease (MASLD). In this study, the methylation levels of those DMRs in liver fibrosis and subsequent HCC were examined.

**Methods:**

The methylation levels of DMRs were investigated using alcoholic cirrhosis and HCC (GSE60753). The data of hepatitis C virus-infected cirrhosis and HCC (GSE60753), and two datasets (GSE56588 and GSE89852) were used for replication analyses. The transcriptional analyses were performed using GSE114564, GSE94660, and GSE142530.

**Results:**

Hypomethylated DMR and increased transcriptional level of zinc finger and BTB domain containing 38 (*ZBTB38*) were observed in HCC. Hypermethylated DMRs, and increased transcriptional levels of forkhead box K1 (*FOXK1*) and zinc finger CCCH-type containing 3 (*ZC3H3*) were observed in HCC. The methylation levels of DMR of kazrin, periplakin interacting protein (*KAZN*) and its expression levels were gradually decreased as cirrhosis progressed to HCC.

**Conclusions:**

Changes in the methylation and transcriptional levels of *ZBTB38*, *ZC3H3*, *FOXK1*, and *KAZN* are important for the development of fibrosis and HCC; and are therefore potential therapeutic targets and diagnostic tools for cirrhosis and HCC.

**Supplementary Information:**

The online version contains supplementary material available at 10.1186/s12876-024-03149-3.

## Background

Hepatocellular carcinoma (HCC) is a major cause of morbidity and mortality worldwide, with an increasing incidence rate [1]. Its most important risk factors are hepatitis B virus (HBV) and hepatitis C virus (HCV). Effective treatments against chronic infections with HBV or HCV have significantly reduced the incidence of viral-associated HCC; however, the incidence of HCC associated with metabolic dysfunction-associated steatotic liver disease (MASLD), formerly known as nonalcoholic fatty liver disease (NAFLD) [2] remains high. Alcoholic steatohepatitis (ASH) is another important risk factor for HCC. Liver fibrosis characterizes disease progression in these chronic liver diseases, and the fibrosis level is a major risk factor for HCC development [3].

MASLD has been prioritized, and genetic and epigenetic analyses have been performed. Our previous genome-wide association studies and those of others have established a definitive genetic background associated with fibrosis stages [4–6]. Genetic variations affect DNA methylation levels and gene expression, indicating that epigenetic changes are important for MASLD development and progression [7]. To evaluate the effect of epigenetic status on fibrosis levels, whole hepatic mRNA sequencing was performed, followed by weighted gene co-expression network analysis (WGCNA) [8]. Two core gene networks associated with MASLD progression were identified, one of which was a scale-free network with four hub genes associated with increased fibrosis and tumorigenesis, while the other was a random network associated with mitochondrial dysfunction. Furthermore, genome-wide hepatic DNA methylation analysis has identified 610 differentially methylated regions (DMRs) associated with fibrosis progression in MASLD [9]. A method to evaluate DMR networks has been developed, and two DMR networks associated with MASLD progression were detected [10]. The methylation levels of DMRs in one of the networks (Network 1) decreased with fibrosis progression. Network 1 included genes involved in transcriptional regulation, cytoskeleton organization, and cellular proliferation and is thus potentially associated with tumorigenesis and fibrosis. Meanwhile, Network 2 was potentially associated with metabolic dysfunction. The methylation levels of DMRs in Network 2 increased with fibrosis progression.

Next, the possible occurrence of DMRs associated with fibrosis in MASLD in other liver diseases, such as viral hepatitis, cirrhosis, and HCC, was investigated. Network 2 was observed in viral hepatitis and HCC, with three potential hub genes: fatty acid binding protein 1 (*FABP1*), serum/glucocorticoid regulated kinase 2 (*SGK2*), and hepatocyte nuclear factor 4 α (*HNF4A*) [11]. *FABP1*, *SGK2*, and *HNF4A* methylation levels in cirrhotic livers were higher than those in normal livers, and their methylation levels in HCC samples were comparable to normal levels. Network 1 was not observed in viral hepatitis or HCC; however, it included zinc finger and BTB domain containing 38 (*ZBTB38*) [12] and formin 1 (*FMN1*) genes [13], which may play important roles in the development of fibrosis and cancer. Thus, it is important to investigate the possible relationship between individual DMRs in Network 1 and liver fibrosis and the potential occurrence of HCC in various chronic liver diseases. In this study, the methylation levels of DMRs in Network 1 in the livers of cirrhosis and HCC patients were examined.

## Methods

### DNA methylation analysis datasets

For the DNA methylation analysis, the hepatic DNA methylation data of normal, alcoholic cirrhosis, and alcoholic HCC (National Center for Biotechnology Information [NCBI] Gene Expression Omnibus [GEO] accession number GSE60753) [14] were used. For replication analyses, hepatic DNA methylation data of normal, HCV-infected cirrhosis, and HCV-infected HCC (GSE60753), those of normal, cirrhosis, and HCC (GSE56588) [15], and those of viral hepatitis and HCC (GSE89852) [16] were used. DNA methylation levels in these datasets were determined using an Infinium HumanMethylation450 BeadChip (Illumina, San Diego, CA, USA). The β-value was used to estimate the methylation level of the CpG locus using the ratio of intensities between methylated and unmethylated alleles.

### Extraction of DMRs associated with both fibrosis and HCC

**Fig. 1:**
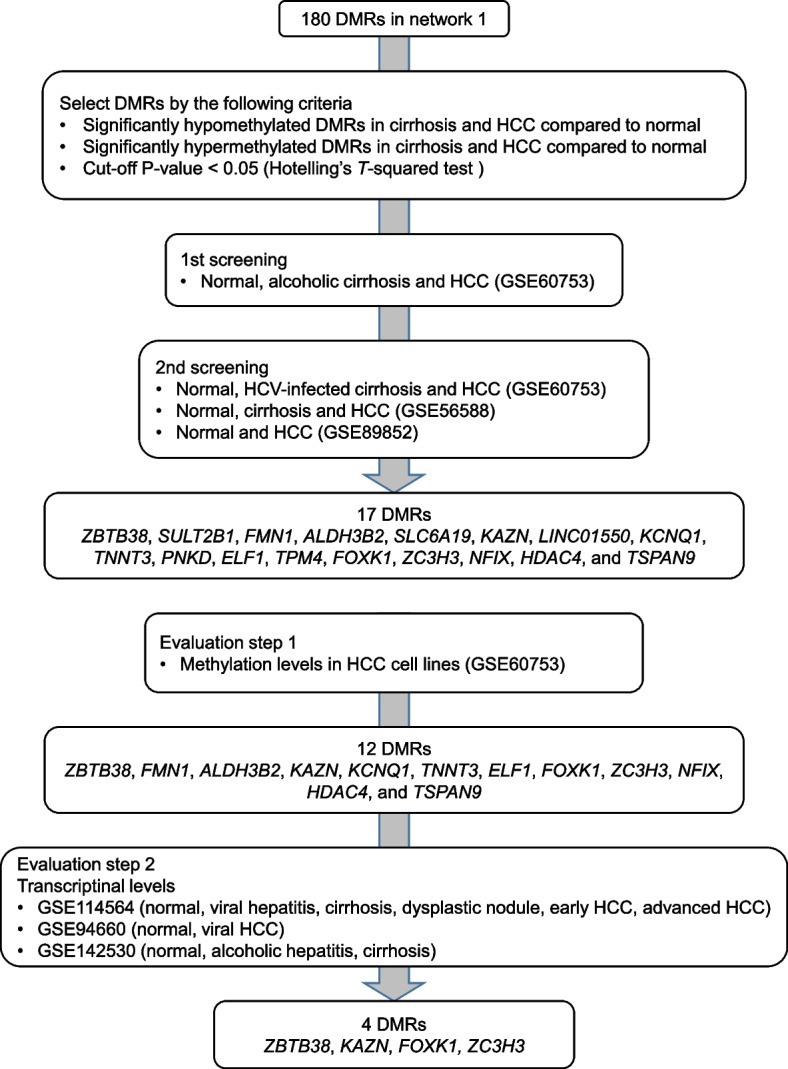
Screening procedures for extracting DMRs related to fibrosis and HCC

The important DMRs associated with both fibrosis and HCC were extracted from 180 DMRs in Network 1, which has been previously reported to be associated with fibrosis progression in MASLD [9, 10]. Screening of DMRs related to fibrosis and HCC was performed as illustrated in Fig. 1. The GSE60753 dataset included methylation levels for 34 normal liver samples, 21 alcoholic and 39 HCV-infected cirrhotic liver samples, and 15 alcohol-related and 12 HCV-infected HCC liver samples [14]. Using this database as the initial screening tool, the methylation levels of DMRs between normal vs. alcoholic cirrhosis and normal vs. alcoholic HCC were compared. This study identified the DMRs associated with both fibrosis and HCC; the change in methylation levels in cirrhosis and HCC should be in the same direction. Significantly hypomethylated or hypermethylated DMRs were extracted from livers with chronic diseases (alcoholic cirrhosis and alcoholic HCC) compared with those from normal livers. For the second screening, the methylation levels of normal, HCV-infected cirrhosis and HCV-infected HCC in GSE60753 were used. In addition, the GSE56588 dataset, which included the data from 11 normal, 10 cirrhotic, and 224 HCC liver samples [15], and the GSE89852 dataset, which included 37 viral hepatitis and 37 HCC liver samples [16] were analyzed. After screening, 17 DMRs related to liver fibrosis and HCC were identified.

### Evaluation of the relationship among 17 identified DMRs, liver fibrosis, and HCC

To confirm whether the 17 identified DMRs were associated with HCC, we examined the methylation levels of normal culture hepatocytes (*n* = 17) and established liver cancer cell lines (*n* = 8) reported in GSE60753) [14]. For further evaluation of these DMRs, we investigated the transcriptional levels using the following RNA sequencing datasets: GSE114564 (15 normal, 20 chronic hepatitis, 10 cirrhosis, 10 dysplastic nodules, 18 early HCC, and 45 advanced HCC liver samples) [17], GSE94660 (21 pairs of tumor and non-neoplastic liver tissues of patients with HBV-HCC) [18], and GSE142530 (12 normal, 10 alcoholic hepatitis, and 6 alcoholic cirrhotic liver samples) [19].

## Statistical analysis

A clustering dendrogram of the samples for the 54 CpG sites in 12 DMRs from the GSE60753 dataset was constructed based on their Euclidean distance using WGCNA [20]. Methylation levels of DMRs between the two groups were evaluated using Hotelling’s *T*-squared test (Hotelling R-package, version 1.0–8). Comparisons of the transcriptional levels between liver disease groups were performed using analysis of variance (ANOVA), along with pairwise comparisons of each liver disease versus normal liver using the *t*-test (ISwR R-package, version 2.0–8).

## Results

### Identification of DMRs associated with both liver fibrosis and HCC

Liver fibrosis is a major cause of HCC development from viral hepatitis, ASH, and MASLD. We have previously reported 610 DMRs associated with fibrosis stages in patients with MASLD [9]. These DMRs were clustered into Networks 1 and 2 [10]. The methylation levels of genes in these networks correlated with increased fibrosis stages in MASLD. Network 2 was persistent in viral hepatitis, cirrhosis, and HCC, with three potential hub genes (*FABP1*, *SGK2*, and *HNF4A*) [11]. Network 1 was not observed in viral hepatitis, cirrhosis, or HCC. The methylation levels of each DMR in Network 1 were strongly associated with fibrosis stages in patients with MASLD; thus, individual DMR in Network 1 was prioritized to examine the possible alterations in its methylation level in liver fibrosis and whether these alterations persist after HCC.

**Fig. 2:**
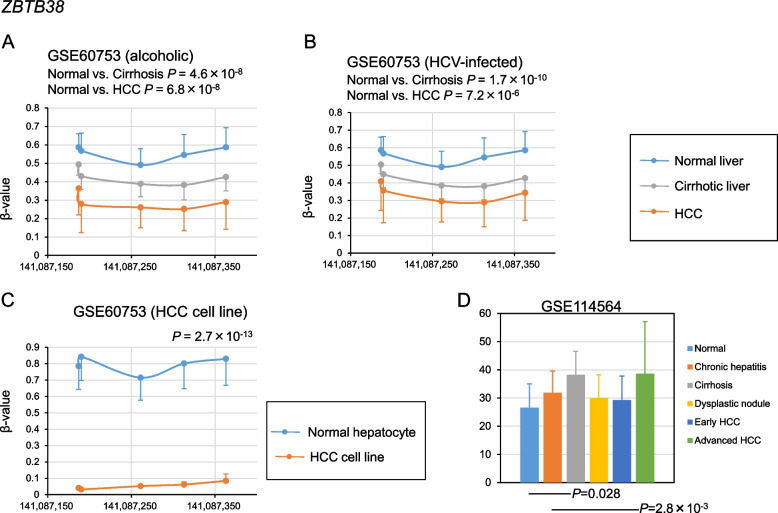
Methylation and transcriptional levels of *ZBTB38* in normal, cirrhotic, hepatocellular carcinoma (HCC) livers, and HCC cell lines. **A** The methylation levels of *ZBTB38* in liver samples from control subjects, patients with alcoholic cirrhosis, and patients with HCC due to chronic alcoholism. *P*-values were calculated using Hotelling’s *T*-squared test. **B** The methylation levels of *ZBTB38* in liver samples from control subjects, patients with HCV-infected cirrhosis, or HCC. *P*-values were calculated using Hotelling’s *T*-squared test. **C** The methylation levels of *ZBTB38* in normal culture hepatocytes and HCC cell lines. *P*-values were calculated using Hotelling’s *T*-squared test. **D** The transcriptional levels of *ZBTB38* in normal, chronic hepatitis, cirrhosis, dysplastic nodule, early HCC, and advanced HCC liver samples. *P*-values were calculated for each disease versus normal liver. Data are expressed as the mean ± standard deviation. Data were analyzed using the GSE60753 and GSE114564 datasets

**Fig. 3:**
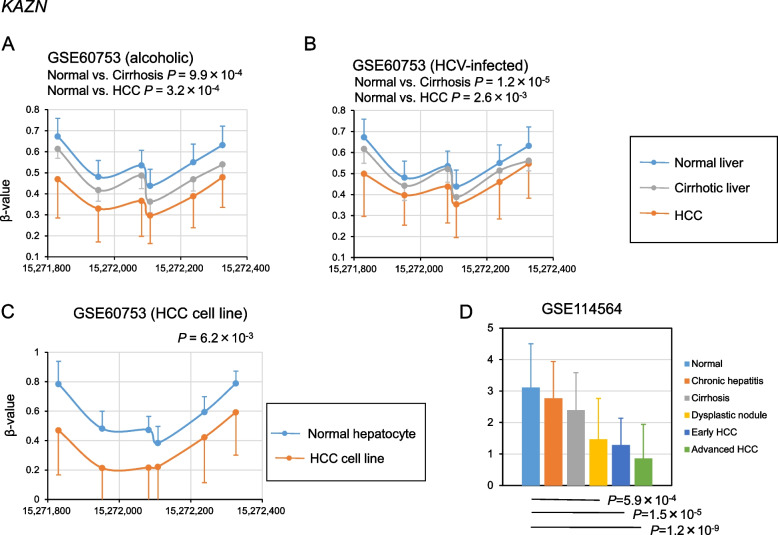
Methylation and transcriptional levels of *KAZN* in normal, cirrhotic, hepatocellular carcinoma (HCC) livers, and HCC cell lines. **A** The methylation levels of *KAZN* in liver samples from control subjects, patients with alcoholic cirrhosis, and patients with HCC due to chronic alcoholism. *P*-values were calculated using Hotelling’s *T*-squared test. **B** The methylation levels of *KAZN* in liver samples from control subjects, patients with HCV-infected cirrhosis, or HCC. *P*-values were calculated using Hotelling’s *T*-squared test. **C** The methylation levels of *KAZN* in normal culture hepatocytes and HCC cell lines. *P*-values were calculated using Hotelling’s *T*-squared test. **D** The transcriptional levels of *KAZN* in normal, chronic hepatitis, cirrhosis, dysplastic nodule, early HCC, and advanced HCC liver samples. *P*-values were calculated for each disease versus normal liver. Data are expressed as the mean ± standard deviation. Data were analyzed using the GSE60753 and GSE114564 datasets

**Fig. 4:**
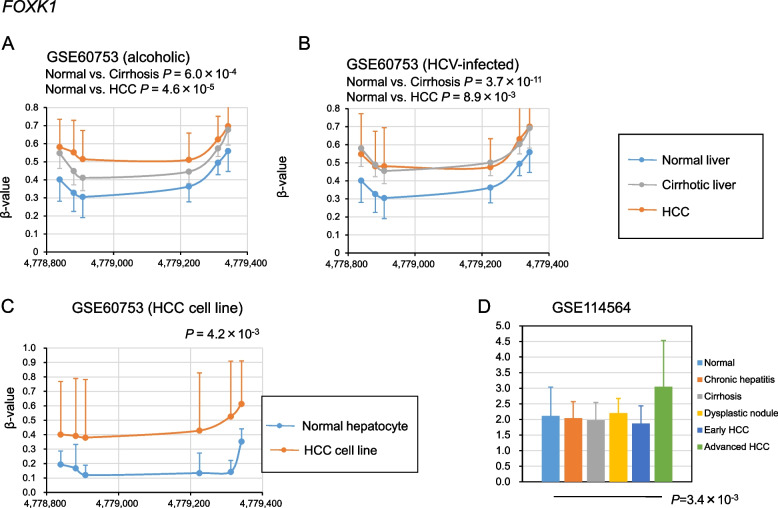
Methylation and transcriptional levels of *FOXK1* in normal, cirrhotic, hepatocellular carcinoma (HCC) livers, and HCC cell lines. **A** The methylation levels of *FOXK1* in liver samples from control subjects, patients with alcoholic cirrhosis, and patients with HCC due to chronic alcoholism. *P*-values were calculated using Hotelling’s *T*-squared test. **B** The methylation levels of *FOXK1* in liver samples from control subjects, patients with HCV-infected cirrhosis, or HCC. *P*-values were calculated using Hotelling’s *T*-squared test. **C** The methylation levels of *FOXK1* in normal culture hepatocytes and HCC cell lines. *P*-values were calculated using Hotelling’s *T*-squared test. **D** The transcriptional levels of *FOXK1* in normal, chronic hepatitis, cirrhosis, dysplastic nodule, early HCC, and advanced HCC liver samples. *P*-values were calculated for each disease versus normal liver. Data are expressed as the mean ± standard deviation. Data were analyzed using the GSE60753 and GSE114564 datasets

**Fig. 5:**
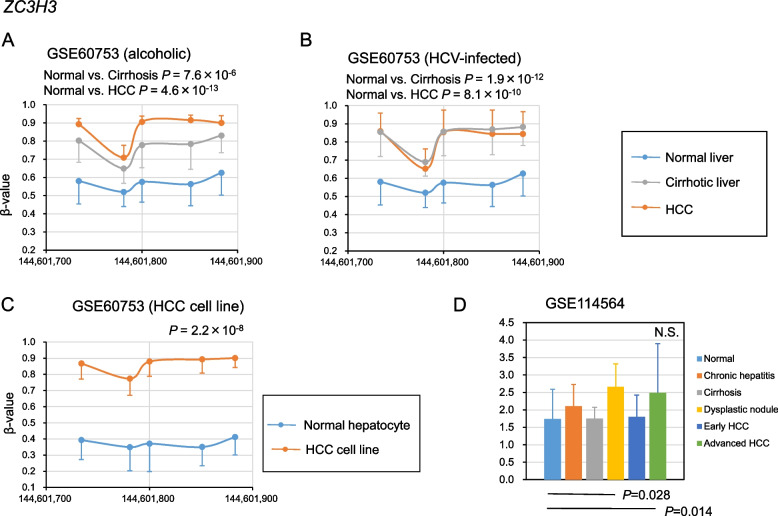
Methylation and transcriptional levels of *ZC3H3* in normal, cirrhotic, hepatocellular carcinoma (HCC) livers, and HCC cell lines. **A** The methylation levels of *ZC3H3* in liver samples from control subjects, patients with alcoholic cirrhosis, and patients with HCC due to chronic alcoholism. *P*-values were calculated using Hotelling’s *T*-squared test. **B** The methylation levels of *ZC3H3* in liver samples from control subjects, patients with HCV-infected cirrhosis, or HCC. *P*-values were calculated using Hotelling’s *T*-squared test. **C** The methylation levels of *ZC3H3* in normal culture hepatocytes and HCC cell lines. *P*-values were calculated using Hotelling’s *T*-squared test. **D** The transcriptional levels of *ZC3H3* in normal, chronic hepatitis, cirrhosis, dysplastic nodule, early HCC, and advanced HCC liver samples. *P*-values were calculated for each disease versus normal liver. Data are expressed as the mean ± standard deviation. Data were analyzed using the GSE60753 and GSE114564 datasets

After screening for DMRs using the method shown in Fig. 1, 17 DMRs were identified, of which 12 were hypomethylated in alcoholic cirrhosis and HCC and 5 were hypermethylated in alcoholic cirrhosis and HCC (Figs. 2, 3, 4, 5, Supplementary Fig. 1). The hypomethylated DMRs observed in alcoholic cirrhosis and alcoholic HCC were found in *ZBTB38*, sulfotransferase family 2B member 1 (*SULT2B1*), *FMN1*, aldehyde dehydrogenase 3 family member B2 (*ALDH3B2*), solute carrier family 6 member 19 (*SLC6A19*), kazrin, periplakin interacting protein (*KAZN*), long intergenic non-protein coding RNA 1550 (*LINC01550*), potassium voltage-gated channel subfamily Q member 1 (*KCNQ1*), troponin T3, fast skeletal type (*TNNT3*), PNKD metallo-beta-lactamase domain containing (*PNKD*), E74 like ETS transcription factor 1 (*ELF1*), and tropomyosin 4 (*TPM4*) genes. Hypomethylated DMRs were confirmed in HCV-infected cirrhosis and HCV-infected HCC, along with two other data sets of cirrhosis and HCC (GSE56588 and GSE89852) (Figs. 2, 3, 4, 5, Supplementary Fig. 1). Hypermethylated DMRs observed in alcoholic cirrhosis and alcoholic HCC were found in forkhead box K1 (*FOXK1*), zinc finger CCCH-type containing 3 (*ZC3H3*), nuclear factor IX (*NFIX*), histone deacetylase 4 (*HDAC4*), and tetraspanin 9 (*TSPAN9*) genes. Hypermethylated DMRs were confirmed in HCV-infected cirrhosis and HCV-infected HCC, as well as in two other data sets of cirrhosis and HCC (GSE56588 and GSE89852) (Figs. 2, 3, 4, 5, Supplementary Fig. 1).

### Methylation levels of the 17 identified DMRs in HCC cell lines

To determine whether cell culture models mimic in vivo methylation changes, we examined the methylation levels of the 17 identified DMRs in HCC cell lines. The datasets GSE60753 includes methylation levels of normal culture hepatocytes (*n* = 17) and established liver cancer cell lines (*n* = 8) [14]. The methylation levels of DMRs in *ZBTB38*, *FMN1*, *ALDH3B2*, *KAZN*, *KCNQ1*, *TNNT3*, and *ELF1* genes were significantly lower in HCC cell lines, while those in *SULT2B1*, *SLC6A19*, *LINC01550*, *PNKD*, and *TPM4* genes did not show significant differences (Figs. 2, 3, 4, 5, Supplementary Fig. 2). The methylation levels of DMRs in *FOXK1*, *ZC3H3*, *NFIX*, *HDAC4*, and *TSPAN9* genes were significantly higher in HCC cell lines (Figs. 2, 3, 4, 5, Supplementary Fig. 2). The methylation levels of 12 DMRs were changed in HCC cell lines as observed in vivo. Further analysis was conducted using these 12 DMRs.

### Transcriptional levels of genes in the 12 DMRs

We investigated the transcriptional levels of the genes in the 12 DMRs and determined whether these DMRs affect transcriptional levels in fibrotic livers and HCC. We used the transcriptional levels from the RNA sequencing datasets GSE114564, which included 15 normal, 20 chronic hepatitis, 10 cirrhosis, 10 dysplastic nodules, 18 early HCC, and 45 advanced HCC liver samples [17]. Replication was performed using RNA sequencing data sets, GSE94660 (21 pairs of tumor and non-neoplastic liver tissues of patients with HBV and/or HCV) [18], and GSE142530 (12 normal, 10 alcoholic hepatitis, and 6 alcoholic cirrhotic liver samples) [19]. The expression of *KAZN* significantly decreased in HCC; notably, *KAZN*’s expression was also decreased in dysplastic nodules but became more pronounced as HCC progressed. The expression levels of the *KAZN* were lower in cirrhosis (Figs. 2, 3, 4, 5, Supplementary Fig. 3). These results suggest that the epigenetic changes *KAZN* are associated with the progression of liver fibrosis and tumorigenesis. The expression levels of the *ZBTB38*, *FOXK1*, and *ZC3H3* were significantly increased in HCC (Fig. 2, Supplementary Fig. 3), while those of *ALDH3B2* and *TNNT3* were too low to evaluate. The expression of *FMN1*, *KCNQ1*, *ELF1*, *NFIX*, *HDAC4*, and *TSPAN9* did not show reproducible significant differences in cirrhosis and HCC (Supplementary Fig. 3).

### Clustering analysis of normal liver and cirrhotic livers from HCV-infected individuals, or chronic alcoholics, and HCC liver samples from HCV-infected individuals or chronic alcoholics

**Fig. 6:**
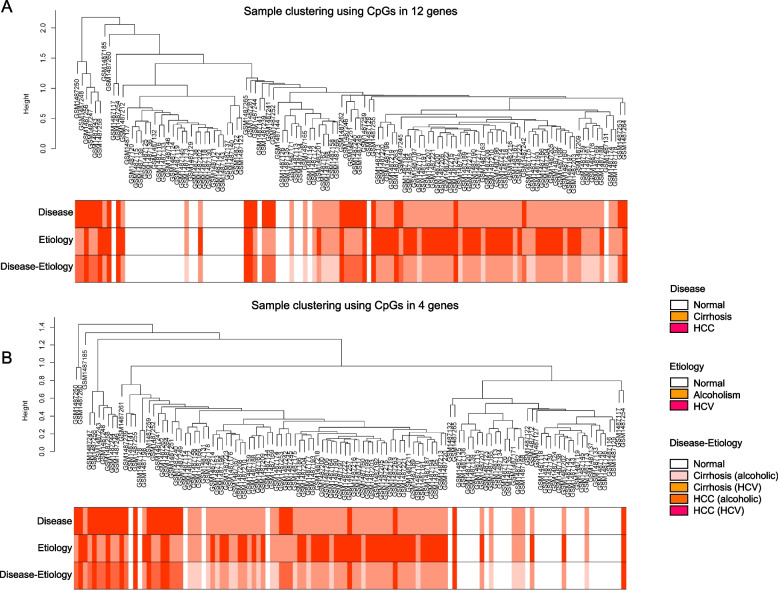
Clustering dendrogram of samples based on their Euclidean distance. Clustering dendrogram of the samples for the 54 CpG sites in 12 differentially methylated regions (DMRs) (**A**) or 22 CpG sites in 4 DMRs (*KAZN*, *ZBTB38*, *FOXK1* and *ZC3H3*) (**B**) retrieved from the GSE60753 dataset and conducted based on their Euclidean distance, using weighted gene co-expression network analysis (WGCNA)

The possibility of using these DMRs to diagnose cirrhosis and HCC was investigated. With clustering analysis using 54 CpG sites in the 12 DMRs, the samples from the GSE60753 dataset [14] were classified accurately into normal, cirrhotic, and HCC liver samples (Fig. 6 A). Using 22 CpG sites in 4 DMRs, namely *KAZN*, *ZBTB38*, *FOXK1*, and *ZC3H3*, also led to the accurate classification of samples (Fig. 6 B). These DMRs did not show a clear classification of etiology, alcoholism, or HCV. The CpG sites consisting of each DMR are listed in Supplementary Table 1.

## Discussion

Liver fibrosis is a major risk factor for HCC development [1, 2]. Epigenetic studies provide a better understanding of the pathogenesis of liver fibrosis, HCC, and various other diseases. Despite the several studies conducted to this effect [14–16, 21, 22], the changes in methylation levels during the progression from liver fibrosis to HCC remain controversial. The methylation levels of multiple consecutive CpG sites affect gene expression; thus, DMR analysis is more effective for epigenetic research [23–25]. We have previously performed DMR analysis using MASLD livers predisposed to HCC and identified 610 DMRs associated with fibrosis stages [9]. These DMRs were clustered into two networks (Networks 1 and 2) [10]. Network 2 contained 430 DMRs and was observed in viral hepatitis and HCC populations with three potential hub genes (*FABP1*, *SGK2*, and *HNF4A*) [11]. DMR methylation level changes observed in liver fibrosis reverted to normal levels in HCC [11]; therefore, the DMRs in Network 2 were not considered strong risk factors for HCC regarding liver fibrosis. Network 1 was not observed in cirrhosis and HCC; however, individual methylation changes were considered important for liver fibrosis and HCC. In this study, 12 DMRs were identified and changes in methylation levels were observed in both liver fibrosis and HCC. The methylation levels of these DMRs were confirmed in HCC cell lines. The methylation levels of 24 CpG sites in four genes could distinguish between normal, cirrhotic, and HCC livers; therefore, DMR analysis in liver diseases is important.

We identified 4 genes, namely, *KAZN*, *ZBTB38*, *FOXK1*, and *ZC3H3*, the epigenetic changes of which are associated with HCC. *ZBTB38*, *FOXK1*, and *ZC3H3* directly participated in epigenetic modifications and their transcriptional levels were increased in HCC. *ZBTB38* is a zinc finger transcription factor (ZNF) that is considered a methyl-CpG binding protein [12, 26]. Its depletion can either promote, reduce, or not affect cell proliferation according to cell type; thus, *ZBTB38* functions as a potential oncogene or tumor suppressor in cancer [27, 28].

*FOXK1* belongs to a family of evolutionarily conserved transcription factors characterized by forkhead DNA-binding domains; it regulates the expression of target genes and contributes to various cellular functions, including the cell cycle, cell growth, proliferation, differentiation, programmed death, metabolism, DNA damage, drug resistance, angiogenesis, and carcinogenesis [29]. *FOXK1* was reported to be upregulated in HCC cells compared with levels in normal liver cells, and its downregulation reduced cell viability [30], consistent with our findings. *ZC3H3* participates in m6A-methyladenine modification, a post-transcriptional regulatory marker in different RNAs, such as messenger RNAs (mRNAs), transfer RNAs (tRNAs), ribosomal RNAs (rRNAs), circular RNAs (circRNAs), micro RNAs (miRNAs), and long non-coding RNAs (lncRNAs). m6A-methyladenine RNA modification is essential in the initiation and progression of human cancers [31, 32]. *ZBTB38* binds to methylation sites and *ZC3H3* catalyzes m6A methylation of RNA, hence, they are involved in epigenetic changes during tumorigenesis. *FOXK1* plays a role in transcriptional regulation. Therefore, increased transcriptional levels of *ZBTB38*, *FOXK1*, and *ZC3H3* may affect the epigenetic regulation of several genes, including their own, leading to the development of HCC.

*KAZN* is a desmosomes component associated with periplakin [33]. We demonstrated that the methylation levels of *KAZN* and its transcriptional levels were decreased in HCC. Indeed, its overexpression stimulates terminal differentiation and reduces cell growth, whereas its knockdown inhibits differentiation and stimulates proliferation [34]. Notably, the methylation levels of *KAZN* were decreased in both cirrhosis and HCC and so were its expression levels as the liver progressed from cirrhosis, dysplastic nodules, and early HCC to advanced HCC. The decreased expression of *KAZN* could inhibit differentiation and stimulate the proliferation of liver cells in cirrhosis, leading to the development of HCC.

The methylation levels of CpG sites in the 12 genes could be used to distinguish between normal, cirrhotic, and HCC livers, which was possible with CpG sites in *KAZN*, *ZBTB38*, *FOXK1*, and *ZC3H3*. The methylation levels of the CpG sites in the 12 or 4 genes could not distinguish between alcoholism- and HCV-derived liver disease. Therefore, these CpG sites could be potentially useful for diagnosing liver cirrhosis and HCC.

## Conclusions

DMR analysis identified 4 genes associated with HCC. Altered methylation and transcriptional levels of *ZBTB38*, *FOXK1*, and *ZC3H3* could deteriorate transcriptional regulation, resulting in the development of HCC. Changes in methylation and transcriptional levels of *KAZN* could alter the cytoskeleton in liver cells, resulting in the development of liver fibrosis and, consequently, HCC. These genes are potential therapeutic targets and diagnostic tools for cirrhosis and HCC.

### Supplementary Information

**Additional file 1: Supplementary Fig. 1.** Methylation levels of 17 genes in normal, cirrhotic, and hepatocellular carcinoma (HCC) livers. GSE60753 (alcoholic): The methylation levels of each gene in liver samples from control subjects, patients with alcoholic cirrhosis, and patients with HCC due to chronic alcoholism. GSE60753 (HCV-infected): The methylation levels of each gene in liver samples from control subjects, patients with HCV-infected cirrhosis, or HCC. GSE56588: The methylation levels of each gene in liver samples from control subjects, patients with cirrhosis, or HCC. GSE89852: The methylation levels of each gene in liver samples from control subjects and patients with HCC. *P*-values were calculated using Hotelling’s *T*-squared test. Data are expressed as the mean ± standard deviation.

**Additional file 2: Supplementary Fig. 2.** Methylation levels of 17 genes in normal and HCC cell lines. The methylation levels of each gene in normal and HCC cell lines. *P*-values were calculated using Hotelling’s *T*-squared test. Data are expressed as the mean ± standard deviation. Data were analyzed using the GSE60753 datasets.

**Additional file 3: Supplementary Fig. 3.** Transcriptional levels of 10 genes in normal, cirrhotic, and hepatocellular carcinoma (HCC) livers GSE114564: The transcriptional levels of each gene in normal, chronic hepatitis, cirrhosis, dysplastic nodule, early HCC, and advanced HCC liver samples. GSE94660: The transcriptional levels of each gene in normal and HCV and/or HBV-infected HCC liver samples. GSE142530: The transcriptional levels of each gene in liver samples from control subjects, patients with alcoholic hepatitis, or alcoholic cirrhotic liver samples. *P*-values were calculated for each disease versus normal liver. Data are expressed as the mean ± standard deviation.

**Additional file 4: Supplementary Table 1.** CpG sites in 17 DMRs associated with liver fibrosis and HCC.

## Data Availability

The data that support the findings of this study are available from NCBI Gene Expression Omnibus (accession number GSE60753, GSE56588, GSE89852, GSE11456, GSE94660, and GSE142530).
