# Supplementary material for: Identification of differentially methylated regions associated with both liver fibrosis and hepatocellular carcinoma
Source: BMC Gastroenterol. 2024 Feb 1;24:57. doi: 10.1186/s12876-024-03149-3 (PMC10832174; doi:10.1186/s12876-024-03149-3)
Supplement: Supplementary file 2 — Additional file 2: Supplementary Fig. 2. Methylation levels of 17 genes in normal and HCC cell lines. The methylation levels of each gene in normal and HCC cell lines. P-values were calculated using Hotelling’s T-squared test. Data are expressed as the mean ± standard deviation. Data were analyzed using the GSE60753 datasets. [file 12876_2024_3149_MOESM2_ESM.docx]

**Supporting Data**

Identification of differentially methylated regions associated with both liver fibrosis and hepatocellular carcinoma

Suguru Kurokawa^1^, Takuro Kobori^1^, Masato Yoneda^2^, Yuji Ogawa^3^, Yasushi Honda^2^, Takaomi Kessoku^2, 4^, Kento Imajo^5^, Satoru Saito^2^, Atsushi Nakajima^2^, and Kikuko Hotta^1^

^1^Laboratoy of Pathophysiology and Pharmacotherapeutics, Faculty of Pharmacy, Osaka Ohtani University, 3-11-1 Nishikiori-kita, Tondabayashi, Osaka 584-8540, Japan

^2^Department of Gastroenterology and Hepatology, Yokohama City University Graduate School of Medicine, 3-9 Fukuura, Kanazawa-ku, Yokohama, Kanagawa 236-0004, Japan

^3^Department of Gastroenterology, National Hospital Organization Yokohama Medical Center. 3-60-2 Harajyuku, Totsuka, Yokohama 245-8675, Japan

^4^Department of Palliative Medicine, International University of Health and Welfare Narita Hospital, 852, Hatakeda, Narita, 286-8520, Japan.

^5^Department of Gastroenterology, Shin-yurigaoka General Hospital. 255 Furusawatsuko, Asao, Kawasaki 2150-0026, Japan

Corresponding author:

Kikuko Hotta, MD, PhD Professor

Laboratory of Pathophysiology and Pharmacotherapeutics, Faculty of Pharmacy, Osaka Ohtani University 3-11-1 Nishikiori-kita, Tondabayashi, Osaka 584-8540, Japan

Tel.: +81-721-24-9423

Fax: +81-721-24-9890

E-mail: [hottakiku@osaka-ohtani.ac.jp](mailto:hottakiku@osaka-ohtani.ac.jp)


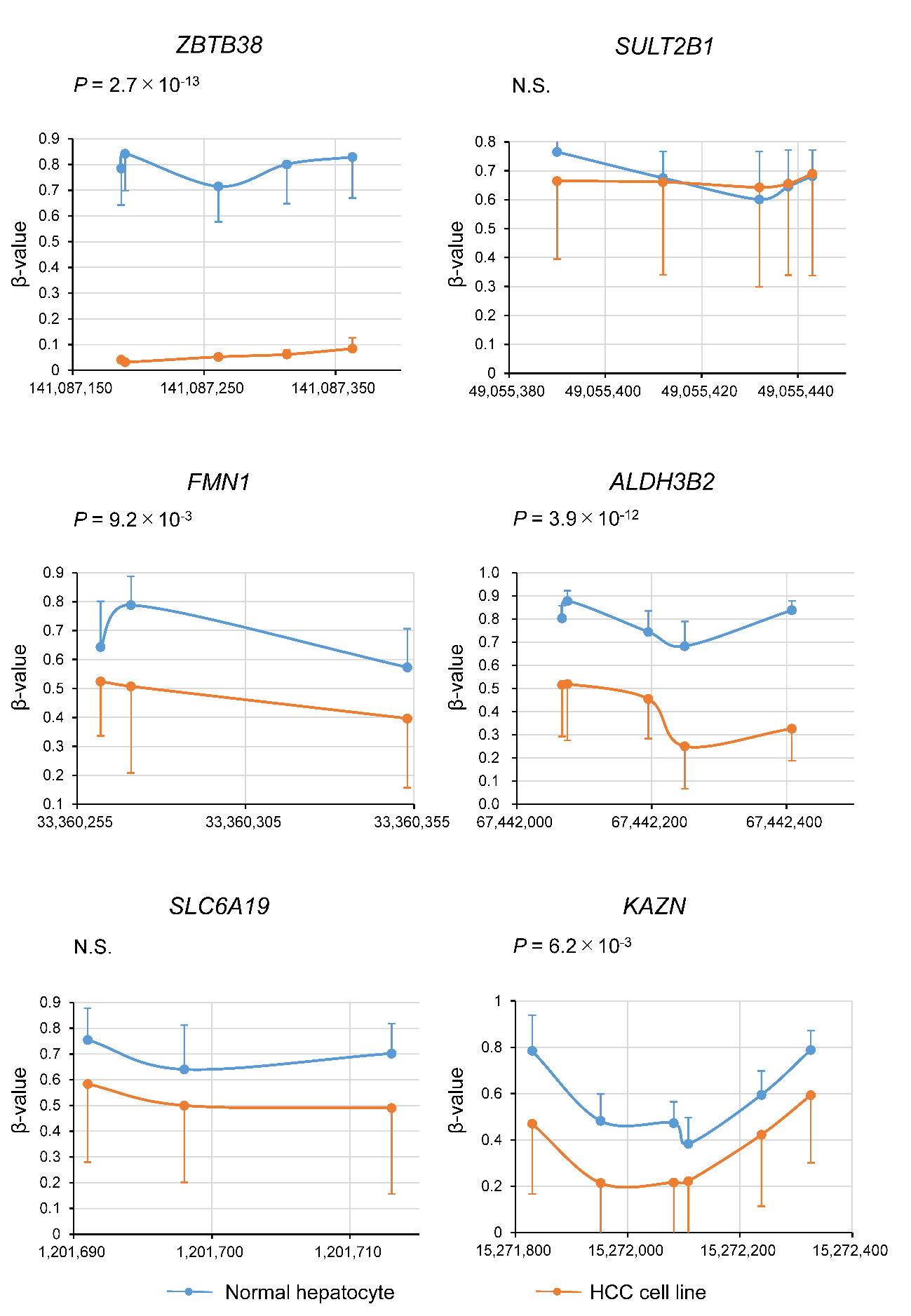


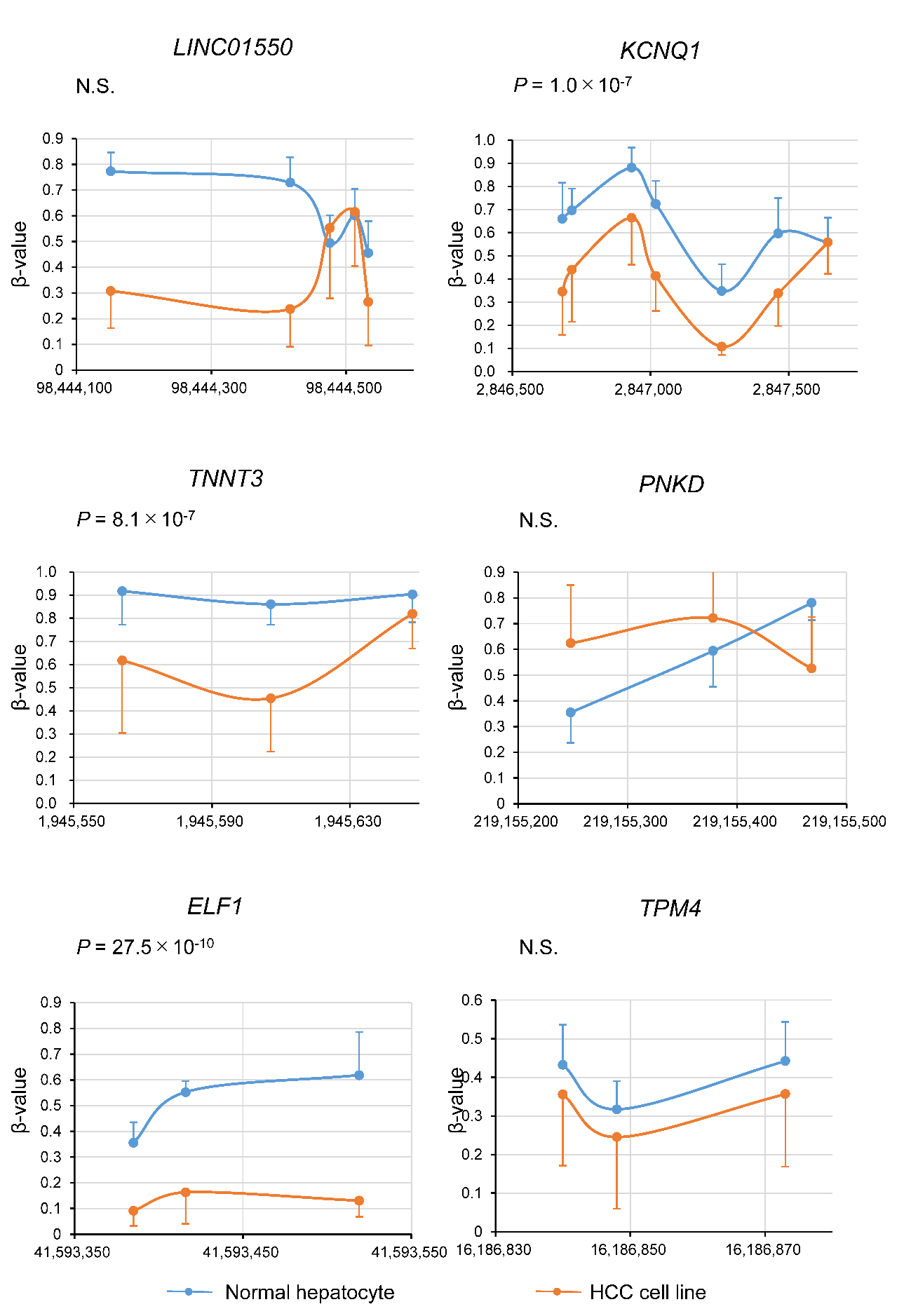


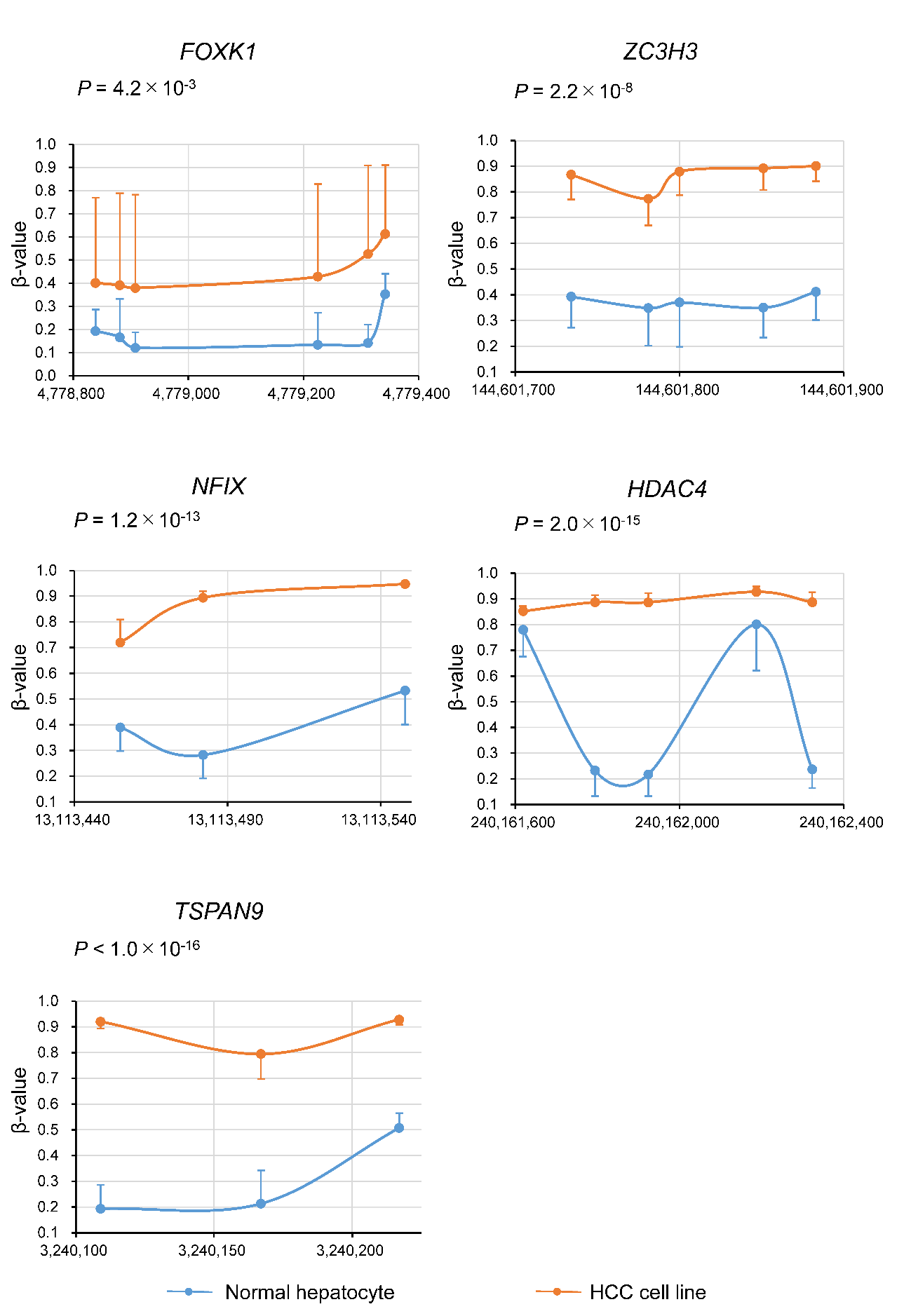


**Supplementary Fig. 2 Methylation levels of 17 genes in normal and HCC cell lines.**

The methylation levels of each gene in normal and HCC cell lines. *P*-values were calculated using Hotelling’s *T*-squared test. Data are expressed as the mean ± standard deviation. Data were analyzed using the GSE60753 datasets.
