# Supplementary material for: Identification of differentially methylated regions associated with both liver fibrosis and hepatocellular carcinoma
Source: BMC Gastroenterol. 2024 Feb 1;24:57. doi: 10.1186/s12876-024-03149-3 (PMC10832174; doi:10.1186/s12876-024-03149-3)
Supplement: Supplementary file 4 — Additional file 4: Supplementary Table 1. CpG sites in 17 DMRs associated with liver fibrosis and HCC. [file 12876_2024_3149_MOESM4_ESM.docx]

**Supplementary Table 1.** **CpG sites in 17 DMRs associated with liver fibrosis and HCC.**

| DMR  (node) | Probe | Chr. | Position  (hg19) | Annotated  genes | CpG feature | |
| --- | --- | --- | --- | --- | --- | --- |
|  |  |  |  |  | Location | Island |
| **ZBTB38** | cg06137072 | 3 | 141,087,187 | *ZBTB38* | 5'UTR | open sea |
|  | cg13029400 | 3 | 141,087,190 | *ZBTB38* | 5'UTR | open sea |
|  | cg08360599 | 3 | 141,087,261 | *ZBTB38* | 5'UTR | open sea |
|  | cg21370924 | 3 | 141,087,313 | *ZBTB38* | 5'UTR | open sea |
|  | cg21474062 | 3 | 141,087,363 | *ZBTB38* | 5'UTR | open sea |
| **SULT2B1** | cg03039843 | 19 | 49,055,390 | *SULT2B1* | TSS200 | open sea |
|  | cg23097961 | 19 | 49,055,412 | *SULT2B1* | TSS200 | open sea |
|  | cg00698688 | 19 | 49,055,432 | *SULT2B1* | 1stExon | open sea |
|  | cg08151612 | 19 | 49,055,438 | *SULT2B1* | 1stExon | open sea |
|  | cg07543967 | 19 | 49,055,443 | *SULT2B1* | 1stExon | open sea |
| **FMN1** | cg15175581 | 15 | 33,360,262 | *FMN1* | TSS200 | open sea |
|  | cg09347959 | 15 | 33,360,271 | *FMN1* | TSS200 | open sea |
|  | cg17454592 | 15 | 33,360,353 | *FMN1* | TSS1500 | open sea |
| **ALDH3B2** | cg20420868 | 11 | 67,442,067 | *ALDH3B2* | 1stExon | open sea |
|  | cg07891457 | 11 | 67,442,075 | *ALDH3B2* | 1stExon | open sea |
|  | cg18492926 | 11 | 67,442,195 | *ALDH3B2* | 5'UTR | open sea |
|  | cg27123351 | 11 | 67,442,249 | *ALDH3B2* | 5'UTR | open sea |
|  | cg24563501 | 11 | 67,442,408 | *ALDH3B2* | TSS1500 | open sea |
| **SLC6A19** | cg02389859 | 5 | 1,201,691 | *SLC6A19* | TSS200 | island |
|  | cg26948274 | 5 | 1,201,698 | *SLC6A19* | TSS200 | island |
|  | cg17650028 | 5 | 1,201,713 | *SLC6A19* | 5'UTR | island |
| **KAZN** | cg00577560 | 1 | 15,271,830 | *KAZN* | Body | open sea |
|  | cg21413173 | 1 | 15,271,952 | *KAZN* | Body | open sea |
|  | cg03398002 | 1 | 15,272,082 | *KAZN* | Body | open sea |
|  | cg16663033 | 1 | 15,272,108 | *KAZN* | Body | open sea |
|  | cg11648522 | 1 | 15,272,238 | *KAZN* | TSS200 | open sea |
|  | cg26577320 | 1 | 15,272,326 | *KAZN* | TSS200 | open sea |
| **LINC01550** | cg00263248 | 14 | 98,444,151 | *LINC01550* | Body | open sea |
|  | cg16062483 | 14 | 98,444,417 | *LINC01550* | Body | open sea |
|  | cg16278496 | 14 | 98,444,476 | *LINC01550* | TSS200 | open sea |
|  | cg11798182 | 14 | 98,444,513 | *LINC01550* | TSS200 | open sea |
|  | cg00034769 | 14 | 98,444,533 | *LINC01550* | TSS200 | open sea |
| **KCNQ1** | cg19852660 | 11 | 2,846,681 | *KCNQ1* | Body | open sea |
|  | cg13859639 | 11 | 2,846,716 | *KCNQ1* | Body | open sea |
|  | cg17416793 | 11 | 2,846,932 | *KCNQ1* | Body | open sea |
|  | cg17333973 | 11 | 2,847,019 | *KCNQ1* | Body | open sea |
|  | cg06485603 | 11 | 2,847,258 | *KCNQ1* | Body | open sea |
|  | cg12141659 | 11 | 2,847,462 | *KCNQ1* | Body | open sea |
|  | cg10151367 | 11 | 2,847,642 | *KCNQ1* | Body | open sea |
| **TNNT3** | cg02556649 | 11 | 1,945,564 | *TNNT3* | Body | shore |
|  | cg02821464 | 11 | 1,945,607 | *TNNT3* | Body | shore |
|  | cg11654118 | 11 | 1,945,648 | *TNNT3* | Body | shore |
| **PNKD** | cg03627290 | 2 | 219,155,248 | *PNKD* | Body | shore |
|  | cg25353281 | 2 | 219,155,378 | *PNKD* | Body | shore |
|  | cg24533564 | 2 | 219,155,468 | *PNKD* | Body | shore |
| **ELF1** | cg01440489 | 13 | 41,593,385 | *ELF1* | 5'UTR | open sea |
|  | cg02632314 | 13 | 41,593,416 | *ELF1* | 5'UTR | open sea |
|  | cg18456803 | 13 | 41,593,519 | *ELF1* | TSS200 | open sea |
| **TPM4** | cg13618979 | 19 | 16,186,840 | *TPM4* | Body | island |
|  | cg11905624 | 19 | 16,186,848 | *TPM4* | Body | island |
|  | cg26968812 | 19 | 16,186,873 | *TPM4* | Body | island |
| **FOXK1** | cg10036013 | 7 | 4,778,839 | *FOXK1* | Body | open sea |
|  | cg03461110 | 7 | 4,778,881 | *FOXK1* | Body | open sea |
|  | cg23752752 | 7 | 4,778,908 | *FOXK1* | Body | open sea |
|  | cg22581896 | 7 | 4,779,225 | *FOXK1* | Body | open sea |
|  | cg01974478 | 7 | 4,779,312 | *FOXK1* | Body | open sea |
|  | cg00208274 | 7 | 4,779,342 | *FOXK1* | Body | open sea |
| **ZC3H3** | cg19598713 | 8 | 144,601,734 | *ZC3H3* | Body | island |
|  | cg18210511 | 8 | 144,601,781 | *ZC3H3* | Body | island |
|  | cg09580859 | 8 | 144,601,800 | *ZC3H3* | Body | island |
|  | cg25900150 | 8 | 144,601,851 | *ZC3H3* | Body | island |
|  | cg00783706 | 8 | 144,601,883 | *ZC3H3* | Body | island |
| **NFIX** | cg19909063 | 19 | 13,113,455 | *NFIX* | Body | island |
|  | cg04646674 | 19 | 13,113,482 | *NFIX* | Body | island |
|  | cg09394128 | 19 | 13,113,548 | *NFIX* | Body | island |
| **HDAC4** | cg04438064 | 2 | 240,161,619 | *HDAC4* | Body | open sea |
|  | cg09634212 | 2 | 240,161,794 | *HDAC4* | Body | open sea |
|  | cg15002163 | 2 | 240,161,924 | *HDAC4* | Body | open sea |
|  | cg04528931 | 2 | 240,162,187 | *HDAC4* | Body | open sea |
|  | cg10094994 | 2 | 240,162,323 | *HDAC4* | Body | open sea |
| **TSPAN9** | cg14129589 |  | 3,240,109 | *TSPAN9* | 5'UTR | open sea |
|  | cg08017323 |  | 3,240,167 | *TSPAN9* | 5'UTR | open sea |
|  | cg09286981 |  | 3,240,217 | *TSPAN9* | 5'UTR | open sea |

Shore: CpG sites existing in up to 2kb from CpG islands; Shelf: CpG sites existing in a region 2–4 kb from CpG islands; Open sea: isolated CpG sites in the genome; TSS1500: the sequence region from –200 to –1,500 bp upstream of the transcription start site (TSS); TSS200: the region from –200 bp upstream of the TSS; 5′UTR: 5′ untranslated region; 3′ UTR: 3′ untranslated region; Body: gene body.
